# Pegylated liposomal doxorubicin (PLD): enhanced skin toxicity in areas of vitiligo

**DOI:** 10.3332/ecancer.2008.111

**Published:** 2008-12-09

**Authors:** Y Yuan, SJ Orlow, J Curtin, A Downey, F Muggia

**Affiliations:** 1 NYU Cancer Institute and Medical Oncology, NYU Langone Medical Center, New York, NY 10016, USA; 2 Department of Dermatology, NYU Langone Medical Center, New York, NY 10016, USA; 3 Department of Gynecologic Oncology, NYU Langone Medical Center, New York, NY 10016, USA

## Abstract

Pegylated liposomal doxorubicin (PLD, Doxil, Caelyx) is widely used for the treatment of ovarian cancer. It is a stable formulation encapsulating doxorubicin in a ‘Stealth’ (i.e., pegylated) liposome with a half-life of about 72 hours. This drastically altered pharmacology confers on it a considerably lower risk of cardiotoxicity, no acute emesis, and near absence of alopecia or problems with extravasation necrosis. On the other hand, PLD's dose-limiting toxicity is cutaneous. Since the original phase I report, cutaneous toxicities reported with PLD fall into four common categories: the well known hand-foot syndrome (also called palmoplantar erythrodysesthesia, or PPE), a diffuse follicular rash, intertrigo-like eruption, and hyperpigmentation including melanotic macules.

## Introduction

Pegylated liposomal doxorubicin (PLD, Doxil, Caelyx) is widely used for the treatment of ovarian cancer. It is a stable formulation encapsulating doxorubicin in a ‘Stealth’ (i.e. pegylated) liposome with a half-life of about 72 hours. This drastically altered pharmacology confers to it a considerably lower risk of cardiotoxicity, no acute emesis and near absence of alopecia or problems with extravasation necrosis. On the other hand, dose-limiting toxicity of PLD is cutaneous [1]. Since the original phase I report [2], cutaneous toxicities reported with PLD fall into four common categories: the well-known hand-foot syndrome (also called palmoplantar erythrodysesthesia, or PPE), a diffuse follicular rash, intertrigo-like eruption and hyperpigmentation including melanotic macules [3].

Pathogenetic mechanisms are in part related to drug or metabolite secretion by sweat glands [4]. Consistent with direct exposure to the drug or metabolites, its severity is lessened with PLD dosing at longer intervals, by regional cooling during drug administration and by drugs such as cisplatin that enhance its clearance [4]. The current observation confirming enhanced toxicity in areas of vitiligo suggests a role for melanin or melanocytes in protecting against PLD-induced erythema and ulceration.

## Case report

A 52-year-old light-skinned woman of Norwegian-English extraction developed Stage IIb fallopian tube cancer in May 2005, and BRCA1 mutation (her mother died at aged 60 after breast and ovarian cancers) was documented. Her surgery consisted of hysterectomy, bilateral salpingo-oophorectomy and omentectomy. She was initially treated with carboplatin plus paclitaxel for six cycles with clinical complete response until November 2006. Upon recurrence she received carboplatin plus gemcitabine followed by intraperitoneal consolidation with cisplatin for five cycles, which was completed in September 2007. In December 2007, with a rapidly rising CA125 and progression of disease in the liver, spleen and pelvic areas, she was started on PLD 40 mg/m^2^ and given two doses four weeks apart before adding oxaliplatin 80 mg/m^2^ three weeks after the second dose due to a ten-fold rise in CA125. One week later she received the third dose of PLD, and this was followed by discomfort in all pre-existing areas of vitiligo, which became deeply erythematous with superficial ulcerations while surrounding areas became deeply hyperpigmented (Figure 1a and 1b). The patient had no excess exposure to sunlight or artificial ultraviolet light during these wintry months. Due to this reaction, the fourth dose of Doxil was held, and she was re-dosed at 30 mg/m^2^ every four weeks together with oxaliplatin. Except for mild erythema, skin changes resolved. Response to the combined treatment, however, only lasted for four months and liver metastases became apparent; she was referred to the National Cancer Institute for consideration for inclusion in trials directed to mutation carriers. However, due to recent exposure to a platinum compound, she was started on a tyrosine kinase inhibitor but further progression in the liver ensued.

## Discussion

Palmoplantar erythrodysesthesia is the most prominent and common dermatologic finding following PLD. In the initial phase I studies, cutaneous reactions were more interval- than dose-dependent and increased in severity more on an every-three-weeks than on an every-four-weeks schedule [2]. The percentage of patients with grade 2–3 skin changes increased, being 60% after the third dose. Studies including fluorescent detection on the skin surface suggest the drug and/or its metabolites are excreted in sweat shortly after PLD infusion is started [5]. This prominent excretion route is consistent with toxicity often observed in intertriginous areas and also in areas where there has been an occlusive dressing or ‘bandaids’ [6] (personal observation). The palmar and plantar surfaces may be more susceptible to PLD skin toxicity in part because of the high density of eccrine sweat glands and/or persistence of a drug in a thick stratum corneum. On the other hand, the preferential toxicity in vitiligo suggests that diminished detoxification by melanocytes or by melanin may play a role. Perhaps melanin affords a mechanism to deal with the effects of free-radicals generated not only upon exposure to ultraviolet light but also to compounds like doxorubicin and its metabolites. A recall phenomenon of sunburn in vitiliginous areas of a patient treated with PLD who denied any recent sun exposure [7] has in fact been reported. The observed toxicity in our patient provides additional support for this increased susceptibility and suggests that protective mechanisms against cutaneous toxicity from PLD may include endogenous melanin production.

## Figures and Tables

**Figure 1a:**
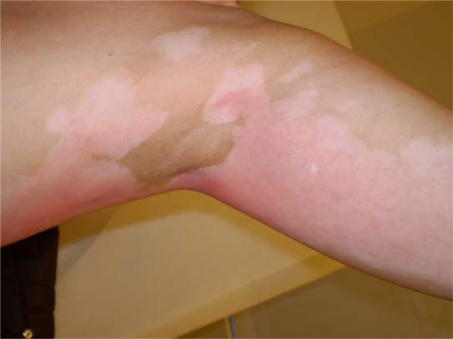
Erythema of pre-existing areas of vitiligo with surrounding hyperpigmented skin changes after doxil infusion

**Figure 1b:**
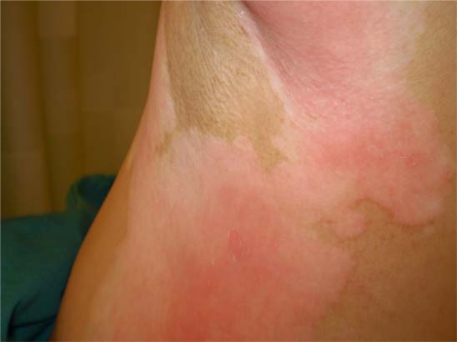
Alternative view of erythema of pre-existing areas of vitiligo with surrounding hyperpigmented skin changes after doxil infusion
